# Nitrous Oxide in Treatment Resistant Major Depression: Should We Laugh About It?

**DOI:** 10.1192/j.eurpsy.2022.1847

**Published:** 2022-09-01

**Authors:** B. Leal, D. Vila-Chã, S. Garcia, I. Pinto, R. Mateiro, M. Avelino, M. Martins, J. Salgado

**Affiliations:** Centro Hospitalar Psiquiátrico de Lisboa, Clínica 1, Lisboa, Portugal

**Keywords:** Depression, Treatment Resistant Major Depression, Nitrous Oxide

## Abstract

**Introduction:**

Nitrous oxide (NO), also known as “laughing gas” is a colorless gas used as an anesthetic, a propellant in some foods, an engine performance enhancer and a recreational drug. When inhaled, it is known to provoke a rapid feeling of euphoria or excitement for a short period of time, dissociative phenomena and sometimes laughter. As its fellow anesthetic agent and NMDA-receptor antagonist, ketamine, NO is being studied for its possible therapeutic profile in treatment resistant major depression (TRMD).

**Objectives:**

TRMD is a serious illness, that urges for effective alternative treatments. In that regard, we explored the recent studies conducted in these patients, using NO in different dosages when compared to placebo.

**Methods:**

The authors revised the published literature about this topic, selecting relevant articles with the topic words: “Depression”, “Treatment Resistant Major Depression” and “Nitrous Oxide” in scientific data base.

**Results:**

Since 2018, at least two randomized clinical trials have demonstrated that NO has considerable antidepressant effects in TRMD, when compared to placebo. Investigators noted that these positive effects where maintained at least for two weeks after a single 1-hour inhalation. In a more recent study, scientists compared different NO concentrations (25% vs. 50%) concluding that the 25% concentration had similar efficacy with a lower risk of adverse effects.

**Conclusions:**

There appears to be encouraging results when treating patients with TRMD with NO in a 25% concentration. Nonetheless, there is need for further investigation, namely through studies that compare NO with other valid TRMD treatments and not only versus placebo.

**Disclosure:**

No significant relationships.

